# Does the Farming Method Influence the Porcine Vomeronasal Organ Condition? A Histological Study

**DOI:** 10.3390/ani14142105

**Published:** 2024-07-18

**Authors:** Violaine Mechin, Pietro Asproni, Eva Teruel, Marion Boutry, Alessandro Cozzi, Patrick Pageat

**Affiliations:** 1 Tissue Biology and Chemical Communication Department, IRSEA—Institute of Research in Semiochemistry and Applied Ethology, 84400 Apt, France; v.mechin@irsea-institute.com (V.M.); marion.boutry30@hotmail.fr (M.B.); 2 Statistics and Data Management Service, IRSEA—Institute of Research in Semiochemistry and Applied Ethology, 84400 Apt, France; e.teruel@irsea-institute.com; 3 Research and Education Board, IRSEA—Institute of Research in Semiochemistry and Applied Ethology, 84400 Apt, France; a.cozzi@irsea-institute.com (A.C.); p.pageat@group-irsea.com (P.P.)

**Keywords:** vomeronasal organ, alteration, pigs, farming condition

## Abstract

**Simple Summary:**

The vomeronasal organ is essential for chemical communication in many species, particularly in pigs, in which the establishment of a social hierarchy in groups is crucial. Alterations to the vomeronasal organ have been shown to be linked to aggressive behaviors in this species. Moreover, it was recently demonstrated in a mouse model that the environmental air composition can impact the condition of the vomeronasal organ. As pigs can be farmed in intensive and free-range farming conditions, the aim of this study was to analyze whether the porcine farming method influences the condition of the vomeronasal organ using a histological approach. The results showed that pigs housed in free-range farming conditions exhibited more alterations in their vomeronasal organs than did those housed in intensive conditions.

**Abstract:**

The vomeronasal organ (VNO) plays a key role in mammals, since it detects pheromones thus enabling social interactions between congeners. VNO inflammatory changes have been shown to severely impact animal life, leading to impaired social interactions in groups, such as in pigs. Environmental air is known to be strongly modified in farms, and it is suspected to be one of the causes of this alteration. This study aimed to compare via histology the VNOs of pigs housed in intensive conditions (n = 38) to those of pigs housed in free-range farming conditions (n = 35). VNO sections were stained in hematoxylin and eosin to assess the presence of nonsensory and sensory epithelium alterations and collagenolysis. The nonsensory epithelium was significantly more inflamed in animals in free-range farming conditions than those in intensive conditions (*p* < 0.0001) and was more strongly affected by signs of collagenolysis (*p* < 0.0001). The sensory epithelium seemed to be less altered by the different environmental conditions (*p* = 0.7267). These results suggest that species-typical pig behaviors, such as digging and rooting for food, could facilitate the presence of microparticles in the oral cavity and their entrance into the vomeronasal canals, leading to changes to the VNO.

## 1. Introduction

The ability of the vomeronasal organ (VNO) to detect chemical signals, thus providing a substantial aspect of social communication, has been well established for several decades [1,2,3]. The key role of this organ has been previously confirmed by the effects induced by its absence or nonfunctionality, which was shown to induce strong behavioral impairments in many species [4,5,6,7,8]. More recently, spontaneous alterations of this organ, such as vomeronasalitis or vomeronasal inflammation in cats [9] and in pigs [10], have also been described, severely impacting the lives of both of these kinds of animals, leading to impaired social interactions. In pigs, an increase in aggressive behaviors above the normal levels usually observed to maintain the dominance hierarchy has been reported. Moreover, this species is known to exhibit intensified aggressive behaviors in intensive farming conditions [11,12]. These stressful conditions are often linked to vulnerability to illness, with a decrease in quality of life and thus meat quality [13]. Therefore, farms have attempted to improve animal living conditions, and free-range farming is currently recognized to improve the quality of life of many species [14,15].

The causes of the increase in aggressive behaviors are numerous, including a reduced ability to detect the social chemical messages exchanged between congeners. Our previous study showed that the presence of spontaneous inflammation in the VNO alters the neuronal layout in pigs, interfering with their chemoreception [16]. The factors responsible for these alterations have not been investigated, although a few hypotheses have been proposed. As reported in our previous studies, environmental conditions strongly influenced the VNO structure in mice reared under laboratory conditions [17]. To date, this effect has not been investigated in real farming conditions.

The objective of this study was to analyze whether environmental air conditions could impact the VNO of pigs, a species commonly reared using two distinct methods: intensive/indoor farming and free-range farming. In humans, exposure to herbicides, solvents, or heavy metals has been described in the respiratory system [18], and air pollution is associated with lung disorders, cerebrovascular diseases, and neuropsychiatric disorders [19,20,21]. In farms with insufficient aeration or intensive production, ammonia is emitted due to the biological degradation of urea present in organic matter (e.g., feces, urine, and bacteria) in the litter [22,23,24,25] and this emission increases with animal density [12,26].

Many studies have investigated the effects of ammonia exposure and have shown that high concentrations of ammonia induce nasal and lung inflammation, weight loss, eosinophil and mononuclear cell infiltration, rhinitis, necrosis, and lesions in the olfactory and respiratory mucosa [12,27,28,29]. Hydrogen sulfides and dust are also found in high quantities in litter and have been shown to damage structures, causing irritation, chronic bronchitis, and epithelial destruction [11,19,26,30,31]. Moreover, behavioral alterations and locomotor dysfunctions were also observed in mice after exposure to house dust mites [32]. These studies suggested the importance of sufficient ventilation in intensive farming conditions and have led to new guidelines for animal farming management.

As previously described, the respiratory system in farm animals is directly impacted by chemical exposure. Although this effect on the olfactory and respiratory epithelium has been well studied, and despite important similarities between this epithelium and the vomeronasal sensory epithelium (VNSE) [2], only one recent study has been conducted on the VNO in a mouse model. This study confirmed an increase in alteration signs in confined conditions with less aeration than in normal housing conditions [17]. This study provided new perspectives for farm animals, such as pigs, where signs of VNO alterations have already been reported [10]. Because pigs are usually reared indoors and under intensive conditions, they could experience more chronic exposure to air contaminants. Indeed, it has already been shown that the presence of environmental contaminants, such as organic dust or natural pollutant gasses (such as ammonia or hydrogen hydroxide) [20,33], which are well represented at farms with high animal densities, is responsible for inflammation and dysfunction of the olfactory epithelium [12,27]. The sensory epithelium of the VNO is structurally and functionally similar to the olfactory epithelium, and the aim of this study was to verify whether the VNO is also impacted by these environmental conditions.

## 2. Materials and Methods

To compare the impact of two different methods of pig farming (free-range and intensive) on the VNO, two groups of pigs were analyzed. Pig snouts from pigs raised in both intensive farming conditions and free-range farming conditions were collected from slaughterhouses. The pigs in the intensive farming group were housed at the Institute of Agrifood Research and Technology (IRTA, Monells, Spain) in pens with slatted floors (5 × 2.7 m) containing one steel drinking bowl connected to a nipple and a concrete feeder with four feeding places. The animals had access to water and food ad libitum. For this group, 38 pig snouts were obtained as part of a previous study [10].

The slaughterhouse in Ales (Alès, France) provided 35 snouts from pigs raised since birth in free-range farming conditions at La Ferme Beauregard (Marguerittes, France). These outdoor conditions included temperature variations (in a Mediterranean climate, ranging from 3 degrees in winter to 31 degrees in summer) and an altitude of 50 m. The pigs had unlimited access to a shelter to protect them from extreme temperatures and a watering place.

Each group included females and castrated males aged 6 to 7 months. The snouts were placed in a 10% formalin solution (pH = 7.4) immediately after death to ensure fixation of the tissues until dissection. Each snout was then opened to collect the two vomeronasal organs. Then, the VNOs were transversally cut to obtain 5 mm paraffin-embedded sections according to routine histological methods. Sections 3.5 µm thick were obtained using a microtome, mounted on slides, dried overnight at 37 °C, deparaffinized with xylene, and rehydrated with alcohol; these sections were used for histological analyses with hematoxylin and eosin (HE) staining (BioOptica, Milan, Italy).

The slides were observed using an inverted microscope (EVOS FL Auto Cell Imaging System; Thermo Fisher Scientific, Carlsbad, CA, USA) to compare their conditions between farming methods and to detect potential differences. The microscopical observations were first performed at magnifications of 4× and 10× to observe the whole VNO in one field, and then at magnifications of ×20 and ×40 to assess the severity and type of inflammation. The observations were repeated on three different transversal sections of each VNO, to obtain a representative picture of their condition. A qualitative description of each VNO was performed to classify the severity of inflammation in the vomeronasal sensory epithelium (VNSE) and in the nonsensory epithelium (NSE) on a scale of 0 to 3 (0 = no sign of inflammation, healthy epithelium; 1 = weak inflammation; 2 = moderate inflammation; and 3 = strong inflammation), as previously reported [10,16].

The presence of aberrant collagen lysis was also evaluated to assess the general condition of the VNO. The degradation of collagen is a physiological process involved in normal tissue maintenance. However, excessive inflammation induces deregulation of the metalloproteinases responsible for the regulation of collagenolysis [34]. This phenomenon has been associated with the progression of different pathological conditions, such as inflammation, cancer metastasis, atherosclerosis, and other diseases [35,36,37].

Finally, the bilaterality of inflammation in the VNSE was also assessed since it has already been shown to be responsible for the occurrence of aggressive behaviors in pig groups [10].

Statistical analysis:

Data analysis was performed using R version 4.2.1 (2022-06-23 ucrt) software and RStudio version 1.4.1103 © (R Foundation for Statistical Computing, Vienna, Austria). The significance threshold was set at 5%.

Two groups of pigs were compared in this study: pigs housed in intensive conditions and pigs housed in free-range farming conditions. Two VNOs (left and right) were collected from each pig. Thus, the animal was included as a random factor in the models. The 4 alteration scores were analyzed: three at the VNO level (VNSE alteration, NSE alteration, and collagenolysis) and one at the snout level (laterality).

All the data were analyzed using ordinal logistic regression (OLR) models, and the random effect of the pig was included for VNSE alteration, NSE alteration, and collagenolysis (MOLR). The assumption of proportional odds was verified with the nominal test.

## 3. Results

### 3.1. Vomeronasal Sensory Epithelium (VNSE) Alterations

**Figure 1:**
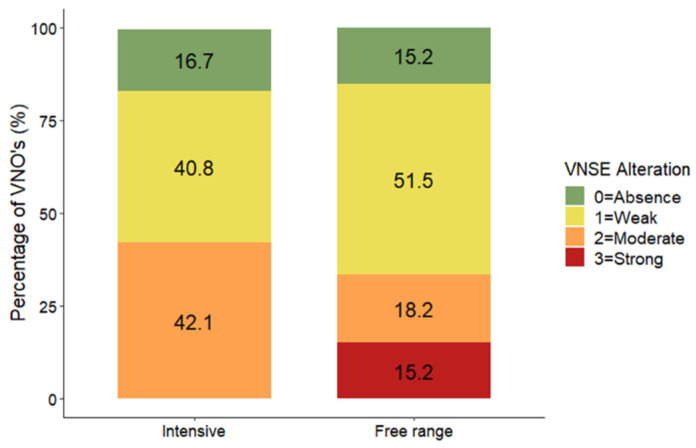
Distribution (%) of vomeronasal sensory epithelium alteration scores between pigs from free-range (N = 66) and intensive (N = 76) farming conditions. The data are shown as the percentage of VNOs assigned to each alteration score.

**Figure 2:**
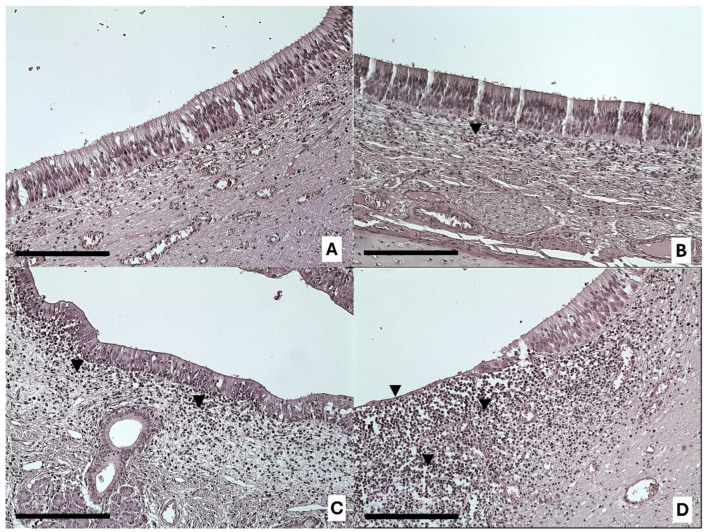
Representative images of inflammation intensities in the VNSE: 0 = healthy epithelium without inflammation (**A**); 1 = weak inflammation (**B**); 2 = moderate inflammation (**C**); and 3 = strong inflammation (**D**), with the presence of inflammatory infiltrations (black arrows). Objective, ×20; scale bar = 200 µm.

Statistical analysis revealed no difference in the VNSE according to the type of farming method (DF = 1; LR = 0.12; *p* = 0.7267; MOLR) (Figure 1 and Figure 2).

### 3.2. Vomeronasal Nonsensory Epithelium (NSE) Alterations

**Figure 3:**
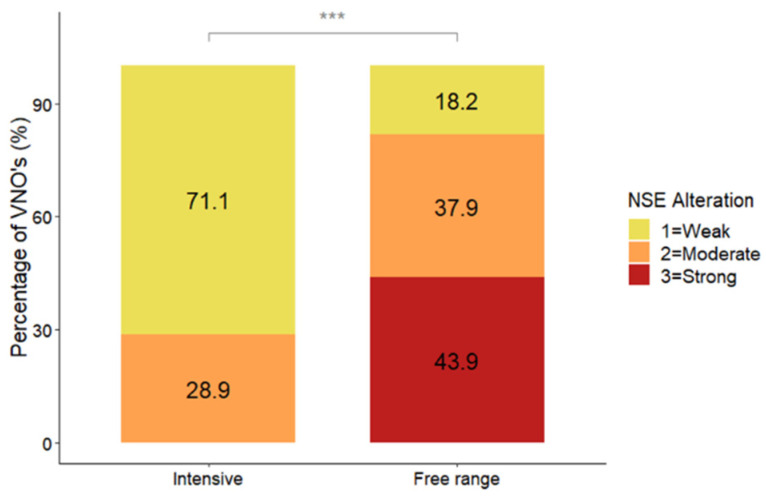
Distribution (%) of vomeronasal nonsensory epithelium alteration scores between pigs from free-range (N = 66) and intensive (N = 76) farming conditions. The data are shown as the percentage of VNOs assigned to each alteration score. *** indicates *p* < 0.001.

**Figure 4:**
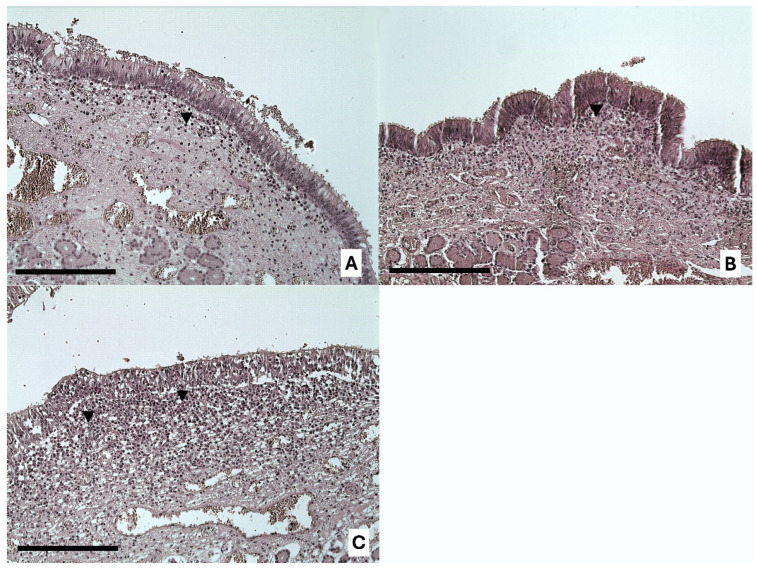
Representative images of inflammation intensities in the NSE: 1 = weak epithelium inflammation (**A**); 2 = moderate infiltration (**B**); and 3 = strong inflammation (**C**), with the presence of chronic inflammatory infiltration (black arrows). Objective, ×20; scale bar = 200 µm.

Statistical analyses revealed that the NSE was more altered in pigs reared in free-range farming conditions than in those reared in intensive farming conditions (DF = 1; LR = 43.57; *p* < 0.0001; MOLR) (Figure 3 and Figure 4).

In both VNSE and NSE inflammation, and in both types of farming systems, the infiltrate was mainly observed in the soft tissue under these epithelia and was of chronic type. In fact, it was mainly composed of mature lymphocytes (Figure 3 and Figure 4), while macrophages and plasma cells were observed in a smaller amount. Moreover, neutrophils were rare and located between epithelial layers, which is a common finding in several epithelia.

### 3.3. Collagenolysis

**Figure 5:**
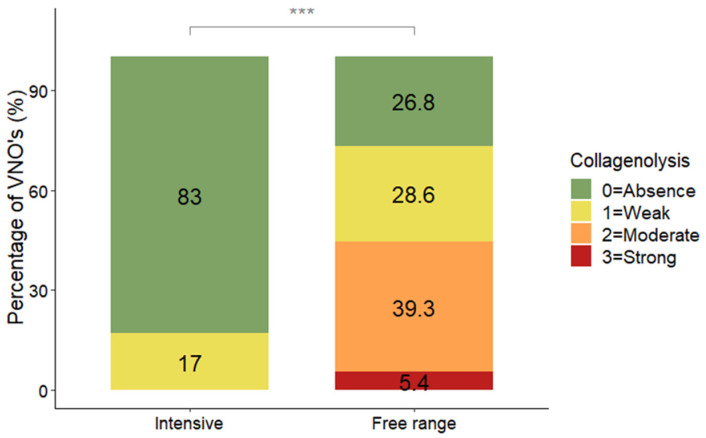
Distribution (%) of collagenolysis scores between pigs from free-range (N = 56) and intensive (N = 42) farming conditions. The data are shown as the percentage of VNOs assigned to each score. *** indicates *p* < 0.001.

Statistical analyses revealed that the VNOs of the pigs reared in free-range farming conditions exhibited more collagenolysis than those of the pigs reared under intensive farming conditions (DF = 1; LR = 36.09; *p* < 0.0001; MOLR) (Figure 5).

### 3.4. Laterality

**Figure 6:**
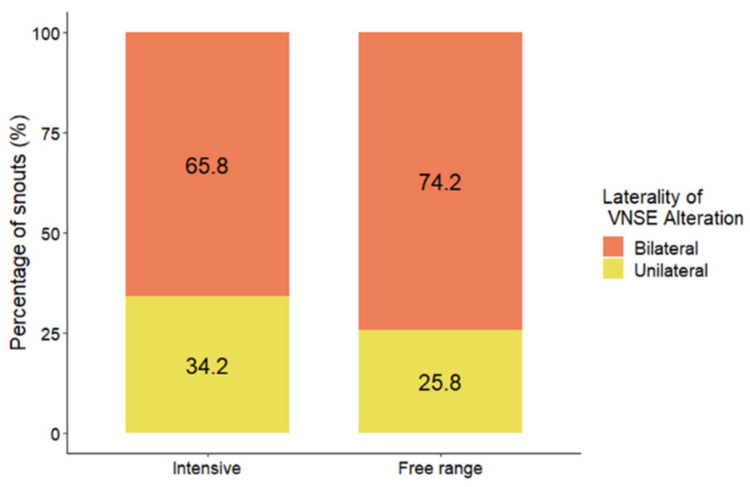
Distribution (%) of inflammation laterality between pigs from free-range (N = 31) and intensive (N = 38) farming conditions. The data are shown as the percentage of snouts with bilateral or unilateral VNSE inflammation.

Statistical analyses showed that farming condition (free-range or intensive) did not lead to differences in the laterality of inflammation in the VNOs of the pigs (DF = 1; LR = 0.57; *p* = 0.4487; MOLR) (Figure 6).

## 4. Discussion

VNO alterations have been described and associated with behavioral changes such as an increase in intraspecific aggressive behaviors in cats [9] and pigs [10], indicating that VNO pathology could lead to a reduction in animal welfare in domestic animals, such as those reared under intensive farming conditions. However, the factors inducing these alterations are still unknown, and elucidating these factors was the aim of this work. Considering that our previous study highlighted the importance of the air composition on the VNO structure in mice in confined environments [17], the aim of this study was to analyze the VNO structure of pigs under real farming conditions. The two main distinct methods of pig farming, intensive/indoor and free-range farming, were compared to determine whether VNO changes could be associated with farming methods.

We found no significant differences in VNSE alteration between the two farming conditions (*p* = 0.7267; MOLR), although descriptive data indicated that strong alterations were found only in animals in free-range farming conditions (15.2%) and not in those in intensive conditions (0%). The NSE exhibited more inflammatory changes in free-range farming conditions than in intensive farming conditions (*p* < 0.0001; MOLR). In our previous experience, the nonsensory epithelium has always been described as having more signs of inflammation than the sensory epithelium in both cats and farm pigs [9,10,16]. Taken together, these findings suggest that the NSE has a protective effect on the whole VNO, reacting strongly and quickly to the presence of external agents (such as dust or other air contaminants). This reaction helps to protect the VNSE and preserve the sensory component, which is crucial for the ability of the animal to detect semiochemicals.

The pigs from the free-range farms were more affected by alterations in collagenolysis than the pigs reared in intensive conditions (*p* < 0.0001; MOLR), with 5.4% of the VNOs classified as having strong collagenolysis and 39.3% classified as having moderate collagenolysis; 0% of the VNOs from pigs from intensive farming exhibited moderate or strong collagenolysis, while 83% were healthy.

Farming conditions did not seem to impact the laterality of VNSE alterations, with no significant differences detected between the groups (*p* = 0.4487; MOLR). This finding is highly important since the bilaterality of VNSE inflammation has previously been linked to an increase in intraspecific aggressive behaviors in farm pigs [10].

In summary, our results suggest that, for some of the tested parameters, pig VNOs seem to be more protected from tissue changes under indoor farming conditions. These findings are consistent with those observed in mice in confined conditions [17], and they seem to confirm the major role of dust and microparticles in the onset of vomeronasalitis in farm pigs. The living conditions of both mice and pigs lead to high exposure to dust: in confined conditions, mice were kept on wood litter (Cat. Aspen: Serlab, Montalaire, France) with reduced ventilation, and in free-range farming conditions, pigs were kept on natural external soil with a high amount of dust and microparticles. Our previous study on mice showed that ammonia only partially increased the presence of VNO changes and that these alterations were mainly induced by living in a confined environment with reduced air ventilation compared with that in normal laboratory housing. We hypothesized that if the main gas (ammonia) was not responsible for the main VNO lesions in the confined individuals, it is likely that the dust produced by the litter played a major role in the onset of these pathological changes. Regarding free-range pig farming, it is plausible that the concentration of contaminant gasses is mitigated by the continuous air movement that leads to their dispersion. On the other hand, the presence of natural soil allows pigs to exhibit species-typical behaviors, such as burrowing and rooting [38]. These behaviors can facilitate the entry of dust and microparticles from the soil into the oral cavity and thus into the vomeronasal canals, which open into the oral cavity. It is well known that these particles are responsible for important changes in the respiratory epithelium [30,39,40]. It is the authors’ opinion that when performing these natural behaviors, the VNOs are more continuously opened than in other situations to detect chemical cues potentially interspersed in the soil, enhancing the entry of these particles. It is well known that the VNO is not constantly open, and its opening is due to an active pumping mechanism under stimulation by different origins [41]. Furthermore, it is also important to consider that the pigs housed in free-range conditions are exposed to larger differences in temperature than are indoor farmed pigs, which could also be a factor inducing alterations in the VNO.

Another important finding of this study is that the bilateral occurrence of VNSE inflammation was not significantly greater in pigs from free-ranging farms than in those from indoor systems. Considering that our previous studies in pigs showed that only bilateral VNSE changes were associated with behavioral issues [10], it is possible that the VNO alterations observed in free-range pigs did not impact their behavior and, consequently, their welfare. Moreover, the free-range system permits pigs to live in lower-density conditions, reducing the risk of conflicts and offering access to different resources and opportunities to exhibit their natural behavior [42], while the higher densities experienced in intensive farming are associated with an increase in social conflicts and aggressive behaviors [43]. From a practical perspective, these findings could provide some interesting suggestions for improving animal welfare, particularly in intensive conditions; for example, this study suggests the benefits of adding straw litter to facilitate natural pig digging behaviors. On the other hand, it is crucial to reflect on the origin of this enrichment matter; for example, favoring matter with low microparticle emissions, such as flax straw, or dust-free litter instead of wheat straw.

Although further studies are needed to confirm this hypothesis, these considerations suggest that the VNO changes described in our study should not have serious impacts on pig behavior and welfare. Finally, our study compared one intensive farm to one free-range farm; therefore, additional research is needed to determine whether this difference is also found in other outdoor farms.

## 5. Conclusions

In conclusion, the results of the present study complement existing knowledge and enable us to identify some of the factors leading to VNO modifications. This study highlighted the role of the environment, dust, and microparticles in VNO alterations and their impact on the life of farm animals such as pigs.

## Data Availability

The data are available upon request.
